# Radiographically Severe but Clinically Mild Reexpansion Pulmonary Edema following Decompression of a Spontaneous Pneumothorax

**DOI:** 10.1155/2014/709560

**Published:** 2014-08-03

**Authors:** William E. Harner, Eric A. Crawley

**Affiliations:** ^1^Department of Internal Medicine, Tripler Army Medical Center, 1 Jarrett White Road, Honolulu, HI 96859, USA; ^2^Department of Pulmonary and Critical Care Medicine, Tripler Army Medical Center, 1 Jarrett White Road, Honolulu, HI 96859, USA

## Abstract

The case is a 48-year-old female who presented with mild dyspnea on exertion and cough with unremarkable vital signs and was found to have a large right sided pneumothorax. She underwent small bore chest tube decompression with immediate reexpansion of the collapsed lung. However, she rapidly developed moderate hypoxemia and radiographic evidence of reexpansion pulmonary edema (REPE) on both the treated and contralateral sides. Within a week, she had a normal chest X-ray and was asymptomatic. This case describes a rare complication of spontaneous pneumothorax and highlights the lack of correlation between symptoms, sequelae, and radiographic severity of pneumothorax and reexpansion pulmonary edema. Proposed pathophysiologic mechanisms include increased production of reactive oxygen species with subsequent loss of surfactant and increased vascular permeability, and loss of vasoregulatory tone.

## 1. Introduction

Reexpansion pulmonary edema (REPE) is a clinical syndrome characterized by the development of clinical and radiographic evidence of pulmonary edema, most commonly seen after large volume (>1.5 L) therapeutic thoracentesis. However, while significantly less common (reported incidence approximately 0.8%) [1, 2] and previously poorly recognized until the advent of routine use of computed tomography, REPE can develop following pneumothorax decompression and is associated with a mortality rate of up to 20% [1]. This is a case of a healthy 48-year-old female who developed REPE following a spontaneous pneumothorax.

## 2. Case Report

**Figure 1:**
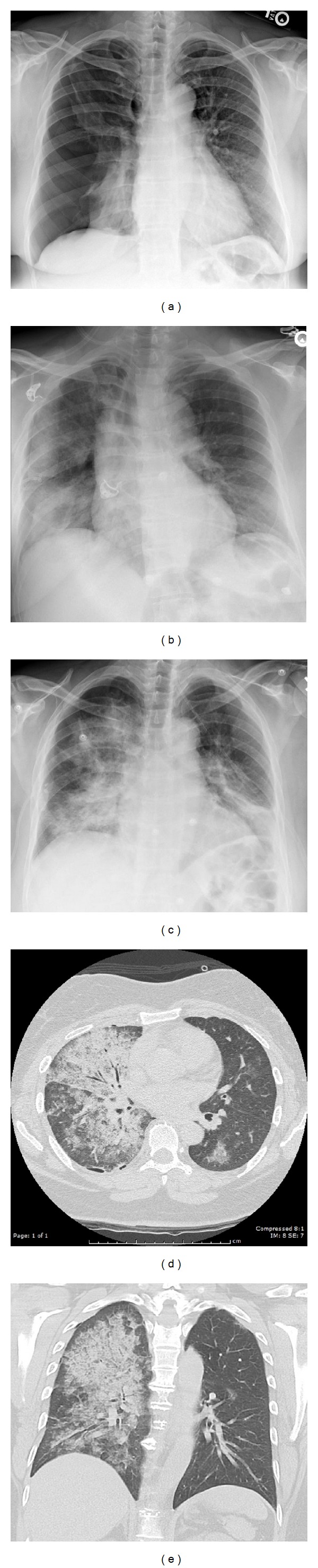
Selected case images. (a) Upright PA/lateral X-ray showing right sided large pneumothorax, (b) portable upright AP X-ray immediate postdecompression, (c) upright PA/lateral showing interval progression of REPE on day 2, (d) representative axial section (expiratory view, high resolution CT), and (e) representative coronal CT reconstruction.

A 48-year-old Pacific Island female with a body-mass index of 35 and a history of a treated latent TB infection presented with four days of a nonproductive cough and mild dyspnea on exertion but no other symptoms or history of trauma. Her vital signs were normal on presentation. A chest X-ray showed a large right sided pneumothorax (Figure 1(a)). She reported a 15-pack-year smoking history and no history of emphysema or chronic bronchitis. She denied any significant family history, including pulmonary or hepatic disease. She was successfully treated with a small bore (“pigtail”) thoracostomy, which was then placed to wall suction (−15 cm H_2_O). Within an hour following thoracostomy, she developed mild hypoxemia with a nadir SpO_2_ of 88%, requiring 3-4 L/min of O_2_ via nasal cannula to maintain adequate oxygenation. She was otherwise asymptomatic. Postprocedure chest X-ray showed near-immediate reexpansion of the right lung but new infiltrates in the right lower and middle lobes (Figure 1(b)); she was then taken off of suction. Computed tomography performed within two hours of thoracostomy placement showed profound ipsilateral pulmonary edema with scattered ground glass opacities in the contralateral lung (Figure 1(d)—axial high resolution expiratory view CT; Figure 1(e)—coronal reconstruction). Despite remaining clinically stable, her radiographic findings visibly worsened on the following morning, with evidence of more definite contralateral involvement (Figure 1(c)). Unilateral pulmonary edema persisted on chest X-ray the following morning; however, she remained clinically stable and the thoracostomy tube was removed later that day. Pulmonary edema resolved by day three without residual symptoms with supportive care alone. Since the patient lacked the typical habitus for spontaneous pneumothorax and the contralateral alveolar filling was significant, further evaluation was done. Cell counts and chemistries, ESR, BNP,* M. pneumoniae* IgM/IgG, *α*_1_-antitrypsin, and HIV ELISA were negative. Only C-reactive protein (4.68 mg/dL) and serum lactate dehydrogenase levels (280 U/L, normal 121–247 U/L) were elevated. On followup at one week, she was asymptomatic and had a normal chest X-ray and normal spirometry. She was referred for polysomnography to rule out obstructive sleep apnea.

The diagnosis of reexpansion pulmonary edema (REPE) was made given the absence of any preexisting findings on chest X-ray, near-immediate onset of pulmonary edema (predominantly ipsilateral to the pneumothorax), and the absence of any evidence of alternative alveolar filling processes (e.g., heart failure and infection). This was further supported by the rapid and complete resolution of her symptoms and radiographic findings.

## 3. Discussion

As discussed, REPE is a rare complication of decompressive thoracostomy for pneumothorax with increasing recognition, while REPE in thoracentesis for effusions can be avoided by termination with the onset of chest discomfort [3], by use of pleural manometry, and routinely by the judicious drainage of pleural fluid [4]; REPE in pneumothorax decompression provides no such early warning. Preventative strategies have not been defined.

Young age, airway obstruction (e.g., OSA) [1], chronicity of the pneumothorax beyond three days (as in this case) [5, 6], mechanism (large versus small bore) and rate of decompression [2, 7], and history of diabetes mellitus [5] have been identified as risk factors for the development of REPE. Trocar and small bore decompression (as used in this patient) carries a higher risk of REPE than traditional hemostat and large bore decompression (odds ratio 5.73)—this is thought to be secondary to the slower release of entrapped air with the slow dissection/hemostat method compared to the single puncture of a trocar assisted thoracostomy [2]. Application of negative pleural pressure may increase the rate of expansion, but this has not been shown to be causative.

Decreased surfactant production, especially with more subacute pneumothoraces, pulmonary nitric oxide levels [2], and rapid increase in cardiac output in the setting of increased pulmonary capillary permeability [8] have been proposed as mechanisms for REPE. Reactive oxygen species (ROS, i.e., free radicals) have been shown to be upregulated following reinflation. In an animal model, these ROS have been shown to trigger xanthine oxidase mediated apoptosis of type II pneumocytes (responsible for surfactant production) and pulmonary vascular endothelium, which may explain increases in permeability and thus the development of REPE [9]. This constellation of risk factors and proposed mechanisms all reflect the rapid increase in blood flow that overwhelms the resting pulmonary arterial tone in the setting of a lower hydrostatic threshold for developing pulmonary edema (from increased permeability) and decreased alveolar surface tension. Changes in flow may be especially problematic in patients that may be predisposed to transient pulmonary hypertension (i.e., OSA). While obstructive apneas and hypopneas can impart transiently very low swings in intrapleural pressures, patients who develop REPE following thoracentesis or decompressive thoracostomy are often awake, which suggest increased awake upper airway resistance or that the chronic changes to pulmonary vascular tone and responsiveness seen in OSA may be to blame. The medical literature is silent on the use of high flow oxygen or positive pressure ventilation as a cause or treatment of REPE. Our patient had no symptoms or radiographic findings to suggest underlying cardiopulmonary disease as a cause or predisposing factor.

It is striking that the radiographic severity of REPE did not correlate with degree of symptoms; however, cases with milder radiographic findings have developed more severe symptoms to the point of hypovolemic shock and severe hypoxic respiratory failure [1]. Given this apparent induction of a hypovolemic state (presumably from increased intravascular volume loss from pulmonary edema), diuretics should be used with caution [10]. Contralateral REPE in spontaneous pneumothorax is a rare finding, having only been described in five other cases, two of which resulted in cardiac arrest [10, 11]. As in this case, the contralateral edema observed in the reported cases was significantly milder than the ipsilateral edema and favored the dependent regions. Bilateral involvement has previously been postulated to be an early form of acute lung injury [10]—this would support a role of inflammatory mediators and loss of surfactant. However, the milder, more dependent localization would suggest regional loss of vasoregulatory tone in dependent portions of the lung (i.e., regions with increased resting blood flow). Management of REPE is supportive with treatment of any resultant hypovolemia and hypoxemia. This case is notable for the radiographic severity of ipsilateral pulmonary edema and evidence of contralateral, but milder, REPE without sequelae.
